# Global burden of low back pain attributable to smoking in 204 countries and territories in 1990–2021

**DOI:** 10.3389/fpubh.2025.1584659

**Published:** 2025-07-10

**Authors:** Baodong Wang, Jiling Zhang, Peng Du, Han Ke, Lei Zang, Shuo Yuan

**Affiliations:** ^1^Department of Orthopedics, Beijing Chaoyang Hospital, Capital Medical University, Beijing, China; ^2^Department of Clinical Laboratory, Beijing Shunyi District Hospital, Beijing, China

**Keywords:** low Back pain, smoking, global burden of disease (GBD), disability-adjusted life years, socio-demographic index

## Abstract

**Background:**

Smoking has a major influence on the development and worsening of low back pain (LBP). However, the effect of smoking on the burden of LBP has not been thoroughly examined at the global, regional, and national levels. This study aims to analyze the trends in smoking-related LBP from 1990 to 2021. It uses data from the 2021 Global Burden of Disease (GBD), providing scientific evidence for policy-making.

**Methods:**

This observational study, based on population data, used epidemiological information on LBP due to smoking from the GBD 2021 study. The study categorized the disability-adjusted life years (DALYs) from smoking-related LBP by year, age, country, and socio-demographic index (SDI). Trends in smoking-related LBP from 1990 to 2021 were assessed using the estimated annual percentage change (EAPC). The GBD frontier analysis was used to monitor and assess progress toward global health goals. Furthermore, the Bayesian age-period-cohort (BAPC) model was applied to predict trends in smoking-related LBP from 2022 to 2036.

**Results:**

In 2021, smoking-related LBP was responsible for 8823.84 × 10^3^ DALYs, a 30.05% increase since 1990. From 1990 to 2021, the age-standardized DALYs rate (ASDR) for smoking-related LBP declined with an EAPC of −1.26%. ASDR dropped in all five SDI regions. Montenegro had the highest DALYs due to smoking-related LBP, while Madagascar saw the greatest decline from 1990 to 2021. Regionally, Central Europe and high-income North America had higher-than-expected LBP burdens, while Western Sub-Saharan Africa, Andean Latin America, Southeast Asia, and Central Latin America had lower-than-expected burdens. Frontier analysis identified countries and regions that need urgent action to lessen smoking-related LBP burden. Finally, it is forecasted that the smoking-related LBP burden will decrease from 2022 to 2036.

**Conclusion:**

From 1990 to 2021, the global ASDR of LBP attributable to smoking decreased, and this downward trend is expected to persist. However, regions with high smoking rates and low socio-economic status still face a significant burden from smoking-related LBP. The data from these regions highlight the need for targeted health policies and interventions to further reduce the burden of smoking-related LBP.

## 1 Introduction

Low back pain (LBP) is a widespread health problem with a high incidence and significant disease burden, greatly affecting individuals’ lives and socio-economic status (1). From 1990 to 2020, the global prevalence and years lived with disability (YLD) rate of LBP declined by 10.4 and 10.5%, respectively. However, the number of individuals affected increased from 552 million to 619 million, indicating a broader reach of the epidemic (2). In regions with a high socio-demographic index (SDI), the burden of LBP is especially prominent, with a disability-adjusted life year (DALY) rate reaching 933.03 per 100,000 individuals in 2021. This highlights the substantial consumption of social health resources. Additionally, high body mass index (BMI), occupational factors, and smoking are major risk factors for LBP. The DALY rate due to high BMI notably increased in 2021, further worsening the disease burden. Projections show that, by 2050, global DALYs due to LBP will rise to 11.63 million, with females bearing a greater burden (3). Treatment of LBP often lacks personalization, failing to adequately consider individual differences among patients, such as etiology, disease course, and psychological state. This results in suboptimal treatment and lower patient satisfaction. Chronic LBP greatly reduces patients’ quality of life, limits their daily activities, and is frequently accompanied by mental health issues, such as anxiety and depression (4). Research shows that around 70% of patients relapse within 12 months after experiencing an episode of LBP, underscoring its chronic and recurrent nature (5). Moreover, LBP is the leading cause of global productivity loss and the main reason for healthy life years lost in 126 countries (6). The direct medical costs and indirect economic losses, including absenteeism, associated with LBP are immense, placing a significant burden on socio-economic conditions (7). This burden is particularly severe in low- and middle-income regions, where limited medical resources further worsen the impact of LBP (8). In summary, as a global health issue, the disease burden and social impact of LBP are substantial and must not be overlooked. Future efforts should aim to improve the prevention and personalized management of LBP, particularly in resource-limited places, by optimizing medical resource allocation, promoting healthy lifestyles, and implementing multi-disciplinary collaborative treatment to alleviate its dual burden on society and individuals.

LBP is a common clinical symptom with a complex etiology, primarily influenced by mechanical, chemical, and psychosocial factors (9). Additionally, multiple studies have confirmed the association between smoking and LBP (10, 11). Nicotine in cigarettes causes vasoconstriction, reducing blood flow to the intervertebral discs, which impairs their nutritional metabolism and accelerates disc degeneration and aging (12, 13). Chronic smoking also ages intervertebral disc collagen, making it more fragile (14). Smoking disrupts estrogen levels, worsens bone loss, and increases the risk of osteoporosis, potentially leading to lumbar instability (15). Furthermore, nicotine inhibits osteoblast activity and promotes osteoclast proliferation, further decreasing bone density (16). Although the global smoking rate declined from 1990 to 2021, the absolute number of smokers has risen due to the increase in population. Tobacco use continues to be a significant public health issue worldwide.

The Global Burden of Disease (GBD) study is an international health research initiative led by the Institute for Health Metrics and Evaluation at the University of Washington, involving over 11,000 researchers from more than 160 countries (17, 18). By measuring the impact of diseases, injuries, and risk factors on human health, the GBD study offers extensive data support for policymakers and public health practitioners (19). Its goal is to provide a complete picture of global health issues and guide health policy and future trend forecasts using key metrics such as all-cause mortality and DALYs. Additionally, the extensive and comparable nature of GBD data enables comparative health analyses across different populations and regions, helping to address health inequalities (20, 21).

However, the global epidemiological pattern of smoking-related LBP burden remains unclear. Thus, we used data from the 2021 GBD study to perform a thorough analysis of DALY trends globally and by country from 1990 to 2021. Additionally, we projected the future disease burden. These findings not only complement existing research but also provide valuable insights for policymakers and healthcare stakeholders, supporting the development of effective strategies and interventions for better strategic planning and policy implementation.

## 2 Methods

### 2.1 Data source

We gathered data on smoking-related LBP from the GBD 2021 Results Tool on the Global Health Data Exchange (GHDx) platform.^1^ The GBD 2021 study compiled information from 100,983 diverse sources, including vital registration systems, cause-of-death analyses, population censuses, household surveys, disease registries, and health service contact records to provide a complete picture of the incidence, prevalence, and DALYs associated with 371 diseases and injuries (22, 23). The study systematically adjusted epidemiological data to address discrepancies from different data sources, definitions, and measurement techniques. Advanced statistical models, including MR-BRT and DisMod-MR 2.1, were used to ensure that the estimates remained consistent across regions, age groups, and genders. By standardizing and calibrating processes, the study reduced the impact of data heterogeneity on the results, offering a high-quality and reliable foundation for global health research (24).

### 2.2 Definition

LBP is defined clinically as pain in the lower back area, ranging from the lower edge of the 12th rib to the level of the gluteal fold. This pain can occur with or without radiating to one or both lower limbs and must last for at least one day. According to the International Classification of Diseases, Ninth Edition (ICD-9), this condition is coded as 724, specifically indicating LBP (25). Additionally, the 2021 Global Health Guidelines define smoking as the current use of any tobacco products or former smokers who have not smoked for at least six months (26).

We chose the DALYs data for smoking-related LBP from the GBD 2021 as our analysis indicator. DALYs combine years of life lost due to premature mortality and YLDs to provide a complete picture of the disease burden, including both premature death and reduced quality of life. This indicator is essential for directly comparing the disease burden across various health concerns and populations, and it serves as an important benchmark for evaluating health inequalities (27).

Moreover, this study uses the SDI as a composite indicator of social development. The SDI is calculated using the geometric mean of three standardized metrics: the total fertility rate among individuals under 25 years old, the average years of education among those aged 15 and older, and per capita lagged income distribution. Calculated at the national level, it accurately reflects the socio-economic development conditions across various countries and regions (28). Based on the SDI, the 2021 GBD study classified 204 countries and regions into five developmental tiers: low SDI, low-middle SDI, middle SDI, high-middle SDI, and high SDI (29).

### 2.3 Statistical analysis

The estimated annual percentage change (EAPC) is a widely used indicator to effectively track trends in key health metrics, including prevalence and incidence, over specific periods (30). This study aims to accurately estimate the dynamic trends of DALYs due to smoking-related LBP from 1990 to 2021. The EAPC is calculated using a regression model, where the year is the independent variable and the natural logarithm of the rate (e.g., prevalence or incidence) of each observation is the dependent variable. A linear regression line is fitted, and its slope is used for the calculation (31). The model’s formula is y = *α* + *β*x + *ε*, where x represents the year, y is the natural logarithm of the rate, α is the intercept, β is the slope, and ε is the random error. The EAPC is then calculated using the formula EAPC = 100 × (exp(β) − 1). The 95% confidence interval (CI) is derived from the fitted regression model. When analyzing the trend results, the range of the CI is important: if the lower limit of the 95% CI is greater than 0, it indicates an upward trend; if the upper limit of the 95% CI is less than 0, it indicates a downward trend; and if the 95% CI includes 0, it indicates that there is no statistically significant change in the trend (32). This study performed a frontier analysis to evaluate the ideal levels of DALYs for 204 countries and regions based on their respective SDI levels (33, 34). It also identifies areas with the largest gaps from these ideal levels. The study used the Bayesian age-period-cohort (BAPC) model to predict global disease burden trends by gender from 2022 to 2036 (35, 36). The BAPC model assumes that the influence of age, period, and cohort are similar across nearby periods. It incorporates Bayesian inference with second-order random walk smoothing to analyze the previous three values and forecast the posterior rates. The model also employs integrated nested Laplace approximation (INLA) to approximate the marginal posterior distributions, avoiding the mixing and convergence issues associated with Markov chain Monte Carlo methods in traditional Bayesian analyses (37). This approach has been widely used in analyzing chronic disease trends and projecting future disease burdens (35, 38). All statistical analyses were conducted using R software (version 4.4.2), with key packages including “BAPC” and “INLA.” In the statistical analyses, a *p*-value of less than 0.05 was considered statistically significant.

## 3 Results

### 3.1 Overview of the global burden

**Figure 1:**
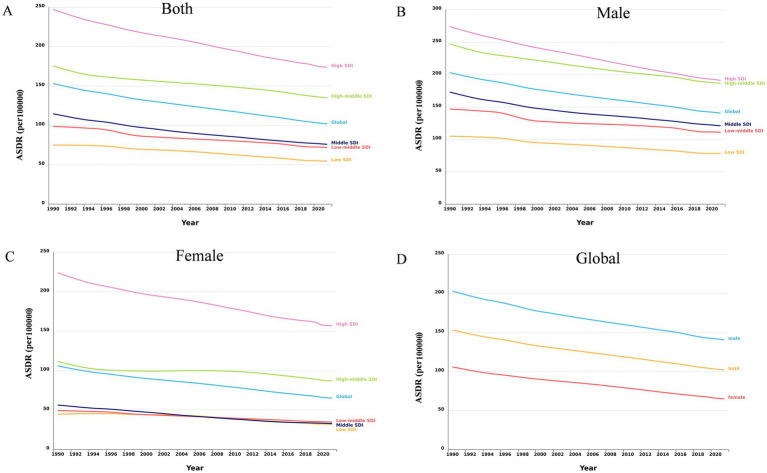
Trends in smoking-attributable LBP ASDR globally and across different SDI regions, 1990–2021. **(A)** The trends of LBP ASDR caused by smoking in both genders globally and in different SDI regions. **(B)** The trend of LBP ASDR caused by smoking among male globally and in different SDI regions. **(C)** The trend of LBP ASDR caused by smoking among female globally and in different SDI regions. **(D)** The global trend of LBP ASDR caused by smoking among male, female, and both genders. DALYs, disability-adjusted life-years; ASDR, age-standardized DALYs rate; LBP, low back pain; SDI, socio demographic index.

From 1990 to 2021, the global number of DALYs due to smoking-related LBP increased by 30.05%, from 6784.85 × 10^3^ in 1990 to 8823.84 × 10^3^ in 2021. However, the age-standardized DALYs rate (ASDR) declined from 153.22 per 100,000 individuals to 102.04 per 100,000 individuals, with a global EAPC of −1.26 (95% CI: −1.28 to −1.24) (Table 1; Figure 1). The ASDR declined for both genders over time, but males continued to experience a significantly higher burden than females (Figure 1). In terms of SDI regions, the highest ASDR in 2021 was found in high SDI regions (247.24 [95% CI 147.56–367.61]), and the ASDR decreased as SDI values declined (Table 1; Figure 1). Among the 21 GBD regions, Eastern Europe was the only region with an increasing EAPC for ASDR (0.41 [95% CI 0.25–0.57]), while all other regions showed a declining trend (Table 1).

**Table 1:** DALYs and ASDR of LBP attributable to smoking in 1990 and 2021 and the temporal trends from 1990.

Characteristics	1990		2021		1990–2021
	NO. × 10^3^ DALYs cases (95% CI)	ASDR per 100,000 (95% CI)	NO. × 10^3^ DALYs cases (95% CI)	ASDR per 100,000 (95% CI)	EAPC no. (95% CI)
Global	6784.85 (4068.10–10067.71)	153.22 (91.36–226.59)	8823.84 (5183.69–13132.64)	102.04 (60.02–152.10)	−1.26 (−1.28 to −1.24)
Socio-demographic index (SDI)					
High SDI	2515.82 (150105–3736.64)	247.24 (147.56–367.61)	2657.54 (1557.55–3985.88)	173.83 (101.73–261.12)	−1.1 (−1.12 to −1.09)
High-middle SDI	1850.64 (1120.97–2718.09)	175.36 (105.26–257.09)	2441.59 (1440.28–3617.69)	135.23 (80.13–200.19)	−0.71 (−0.75 to −0.68)
Middle SDI	1456.28 (878.33–2165.74)	114.81 (69.15–170.11)	2164.40 (1269.18–3199.77)	76.01 (44.67–112.12)	−1.3 (−1.34 to −1.26)
Low-middle SDI	739.93 (436.13–1099.51)	98.84 (58.37–146.14)	1190.69 (682.36–1784.65)	71.99 (41.26–107.69)	−1.02 (−1.08 to −0.97)
Low SDI	211.63 (124.14–318.12)	75.09 (44.42–112.86)	358.37 (204.50–550.97)	54.55 (31.26–83.62)	−1.1 (−1.15 to −1.05)
GBD region					
High-income Asia Pacific	444.22 (269.45–659.21)	217.03 (131.36–320.48)	409.16 (239.09–620.83)	149.11 (87.65–223.84)	−1.28 (−1.36 to −1.21)
Central Asia	63.91 (38.07–94.53)	121.03 (72.40–179.49)	115.70 (68.87–171.12)	118.80 (70.44–176.30)	0.06 (−0.03–0.15)
East Asia	1342.39 (820.83–1974.41)	130.61 (79.52–191.32)	1931.90 (1133.67–2820.26)	91.53 (53.83–132.11)	−0.99 (−1.08 to −0.9)
South Asia	675.79 (399.29–1003.32)	94.50 (56.27–139.92)	952.98 (532.38–1449.59)	57.54 (32.28–87.03)	−1.61 (−1.7 to −1.52)
Southeast Asia	274.98 (163.74–404.52)	86.70 (51.76–126.73)	542.50 (320.25–799.19)	72.60 (43.01–106.54)	−0.62 (−0.64 to −0.59)
Oceania	4.62 (2.68–6.90)	110.35 (63.89–165.27)	11.45 (6.66–17.62)	105.08 (61.59–161.96)	−0.21 (−0.25 to −0.18)
Australasia	58.99 (34.86–88.50)	262.80 (155.70–394.40)	70.88 (40.35–107.85)	180.54 (102.35–276.26)	−1.19 (−1.24 to −1.14)
Central Europe	484.36 (293.52–719.41)	335.42 (202.65–495.56)	474.49 (281.09–708.90)	287.97 (172.35–428.50)	−0.46 (−0.48 to −0.45)
Eastern Europe	488.26 (296.57–718.61)	183.70 (110.67–270.14)	566.73 (341.67–837.63)	194.02 (115.52–290.13)	0.41 (0.25–0.57)
Western Europe	1120.54 (675.55–1667.64)	237.44 (143.19–354.51)	1163.97 (684.40–1753.47)	186.92 (110.59–281.44)	−0.72 (−0.73 to −0.7)
Andean Latin America	12.66 (7.11–19.73)	51.34 (28.96–79.32)	29.55 (16.94–45.60)	46.11 (26.33–71.07)	−0.36 (−0.39 to −0.32)
Central Latin America	102.19 (59.36–155.60)	95.14 (55.59–142.86)	158.76 (90.20–244.60)	59.74 (33.96–92.14)	−1.64 (−1.73 to −1.55)
Southern Latin America	104.77 (61.29–153.68)	223.17 (130.14–327.44)	146.90 (83.61–219.85)	188.01 (107.02–281.97)	−0.63 (−0.7 to −0.56)
Tropical Latin America	233.40 (137.43–353.06)	203.36 (119.58–307.23)	307.38 (171.46–468.67)	115.91 (64.73–176.83)	−2.10 (−2.20 to −2.00)
Caribbean	27.68 (16.02–42.46)	98.69 (56.84–151.26)	38.10 (21.55–58.40)	72.13 (40.82–110.62)	−1.11 (−1.22 to −0.99)
High-income North America	900.30 (534.25–1342.83)	284.09 (168.24–424.33)	934.24 (547.02–1407.63)	191.56 (112.54–289.93)	−1.17 (−1.23 to −1.12)
North Africa and Middle East	281.55 (168.26–422.49)	130.25 (77.73–194.04)	664.83 (391.01–995.03)	111.97 (65.47–166.69)	−0.45 (−0.48 to −0.43)
Central Sub-Saharan Africa	16.78 (9.55–25.74)	55.15 (31.30–84.40)	40.19 (23.42–62.51)	48.68 (28.33–74.09)	−0.31 (−0.43 to −0.19)
Eastern Sub-Saharan Africa	62.81 (36.35–95.06)	66.33 (38.56–100.72)	122.30 (69.90–184.62)	52.20 (29.47–79.84)	−0.83 (−0.87 to −0.78)
Southern Sub-Saharan Africa	37.66 (22.15–56.71)	116.90 (68.47–176.32)	50.10 (28.87–77.21)	70.45 (40.54–108.15)	−1.6 (−1.72 to −1.47)
Western Sub-Saharan Africa	46.88 (26.44–72.31)	42.19 (23.92–64.59)	91.62 (51.47–142.82)	32.65 (18.28–50.65)	−0.92 (−1.04 to −0.79)

NO. × 10^3^ DALYs, number×10^3^ disability-adjusted life-years; EAPC, estimated annual percentage change; CI, confidential interval; DALYs, disability-adjusted life-years; ASDR, age-standardized DALYs rate; SDI, socio demographic index.

**Figure 2:**
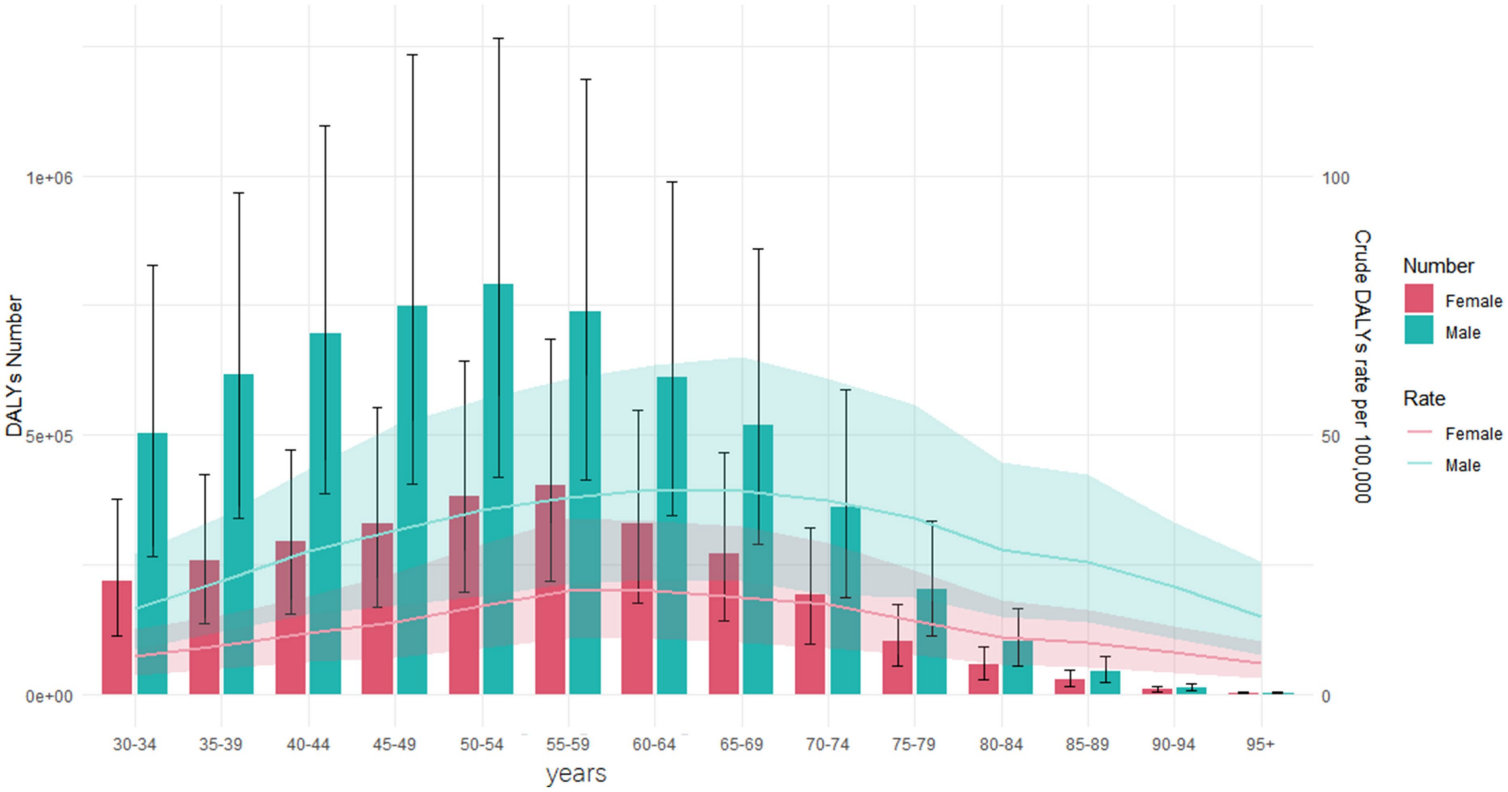
DALYs number and crude DALYs rate for smoking-induced LBP by age and sex worldwide in 2021. DALYs, disability-adjusted life-years; LBP, low back pain.

**Figure 3:**
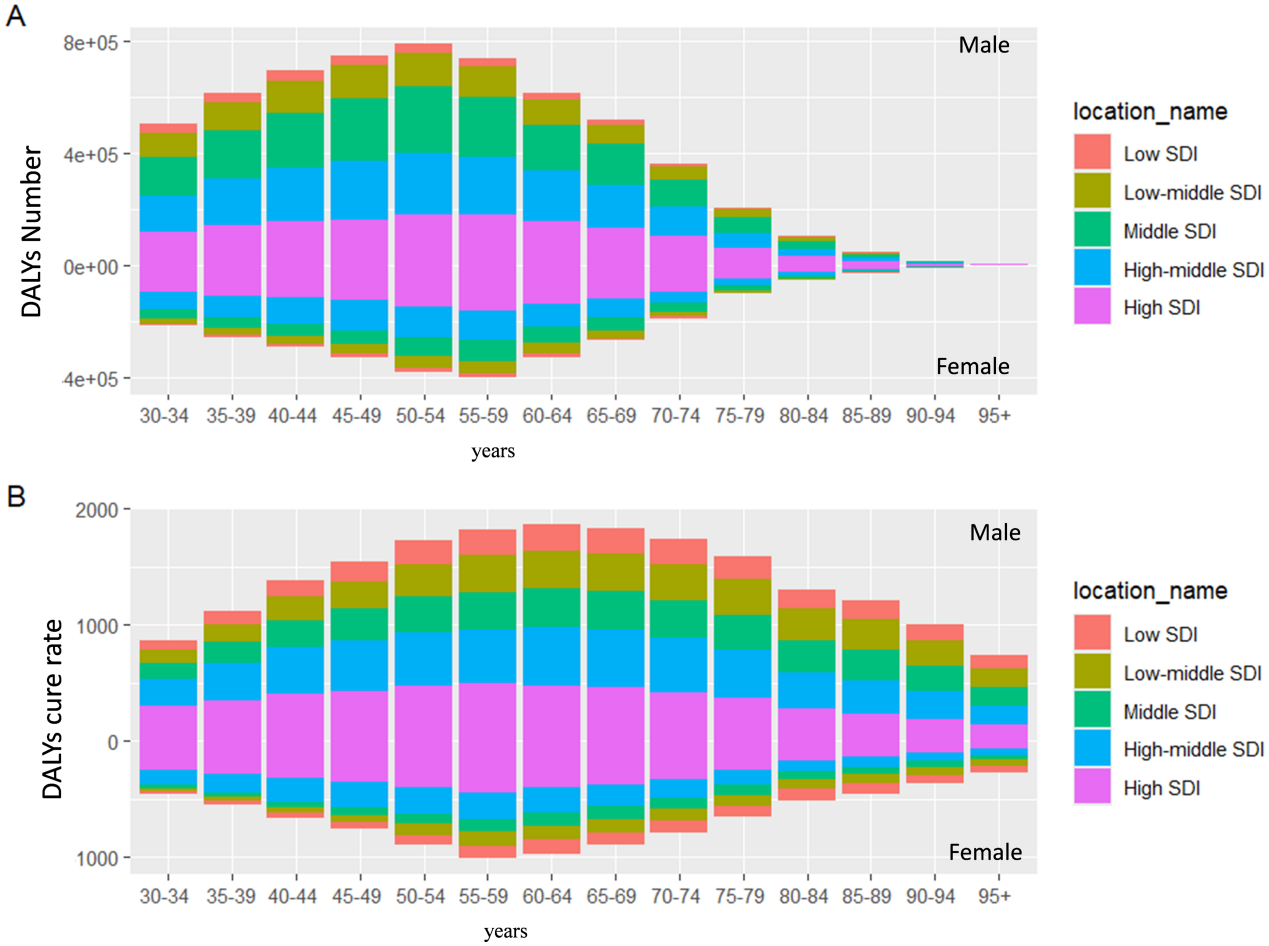
The number of DALYs and crude DALYs rate of smoking-induced LBP in different SDI by age and sex. **(A)** DALYs number. **(B)** Crude DALYs rate. DALYs, disability-adjusted life-years; LBP, low back pain; SDI, socio demographic index.

Figure 2 highlights the differences in the distribution of DALYs across various age groups worldwide in 2021. The age group with the highest number of DALYs was 40–44 years, while the highest ASDR was seen in the 60–64 years age group. In all age groups, males had a higher burden than females. In 2021, countries with low, middle-low, and middle SDI displayed an approximately normal distribution of DALYs across various age groups, whereas other regions showed a skewed distribution. Additionally, from 1990 to 2021, the crude DALYs rate across all age groups significantly declined in all SDI regions (Supplementary Figure 1). In high SDI regions, the highest DALYs rate for both females and males was in the 55–59 years age group. Conversely, in middle-low SDI regions, the peak DALYs rate was in the 50–54 years age group for females and in the 60–64 years age group for males (Figure 3).

### 3.2 Global appraisal of the disease burden of LBP due to smoking

**Figure 4:**
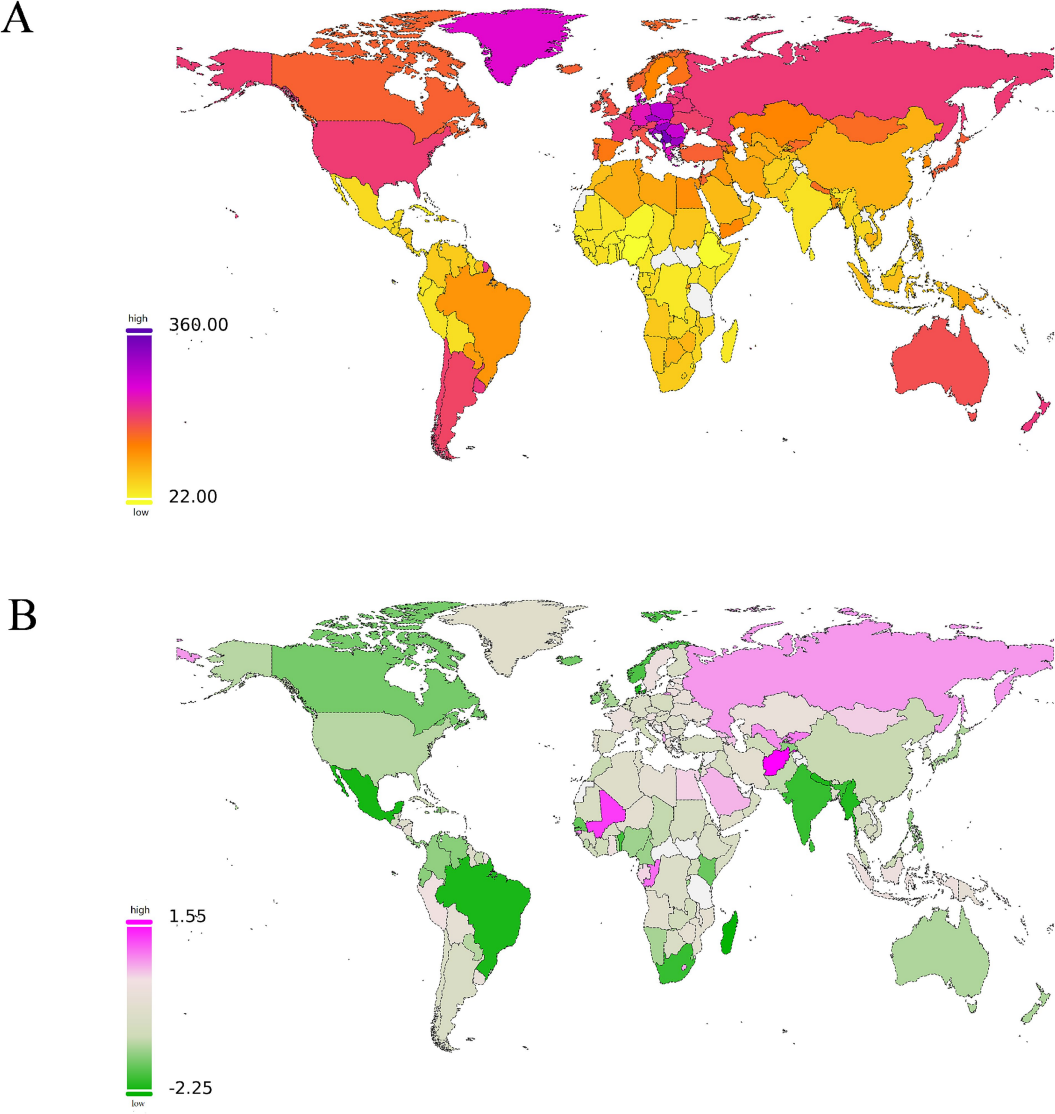
The manifestations and changes of the global disease burden of low back pain (LBP) attributable to smoking across 204 countries and regions. **(A)** The spatial distribution of LBP ASDR attributable to smoking in 2021. The burden gradually changes from yellow to orange, then to pink from low to high, and the highest is represented by purple. **(B)** The EAPC in LBP ASDR attributable to smoking from 1990 to 2021. Green represents descent. The darker the color, the greater the descent. Red represents ascent. The darker the color, the greater the descent. DALYs, disability-adjusted life-years; ASDR, age-standardized DALYs rate; EAPC, estimated annual percentage; LBP, low back pain; SDI, socio demographic index.

The world map shows the ASDR of smoking-related LBP and the corresponding EAPC for different countries and regions in 2021 (Figure 4). Notably, several countries in Southeastern Europe carry a significant burden, with Montenegro showing the highest figures. Additionally, some Central and Western European countries, such as Slovenia and Denmark, report higher ASDR (Figure 4A). From 1990 to 2021, more than 80% of countries have experienced a decrease in EAPC values to varying degrees, with Madagascar and Mexico registering the most significant reductions. In contrast, a few countries, such as Afghanistan, have seen an increased burden (Figure 4B; Supplementary Table 1).

### 3.3 Trends in SDI regions or countries

**Figure 5:**
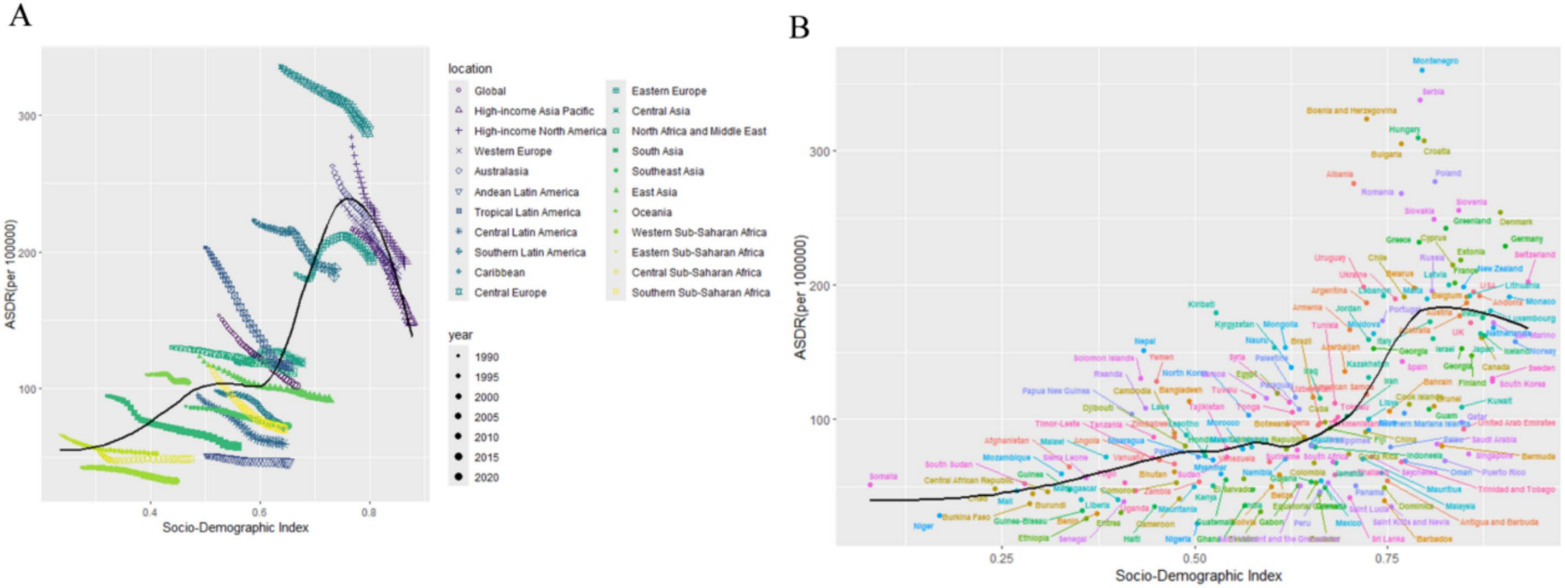
Association between age-standardized LBP attributed to smoking DALYs and SDI. **(A)** Trend in ASDR of LBP attributed to smoking among/across 21 regions and global based on SDI in 2021. **(B)** Trend in ASDR of LBP attributed to smoking among/across 204 countries based on SDI in 2021. DALYs, disability-adjusted life-years; ASDR, age-standardized DALYs rate; LBP, low back pain; SDI, socio demographic index.

As economic levels improve, the ASDR burden of smoking-related LBP has generally shown an upward trend. Across 21 regions, the burden of smoking-related LBP increases slowly when the SDI is below 0.5. When the SDI ranges from 0.5 to 0.6, the ASDR burden remains fairly stable. The burden accelerates and peaks at an SDI of 0.75 when the SDI is between 0.6 and 0.75. Once the SDI exceeds 0.75, the burden begins to decline gradually. Regionally, Central Europe and high-income North America report LBP burdens that are higher-than-expected levels. In contrast, regions such as Western Sub-Saharan Africa, Andean Latin America, Southeast Asia, and Central Latin America show lower than anticipated burdens (Figure 5A). At the country level, nations such as Montenegro, Serbia, Bosnia and Herzegovina, and Hungary experience higher-than-expected smoking-related LBP burdens. Conversely, Barbados, Puerto Rico, and Nigeria report burdens that are lower than anticipated (Figure 5B).

### 3.4 Frontier analysis

**Figure 6:**
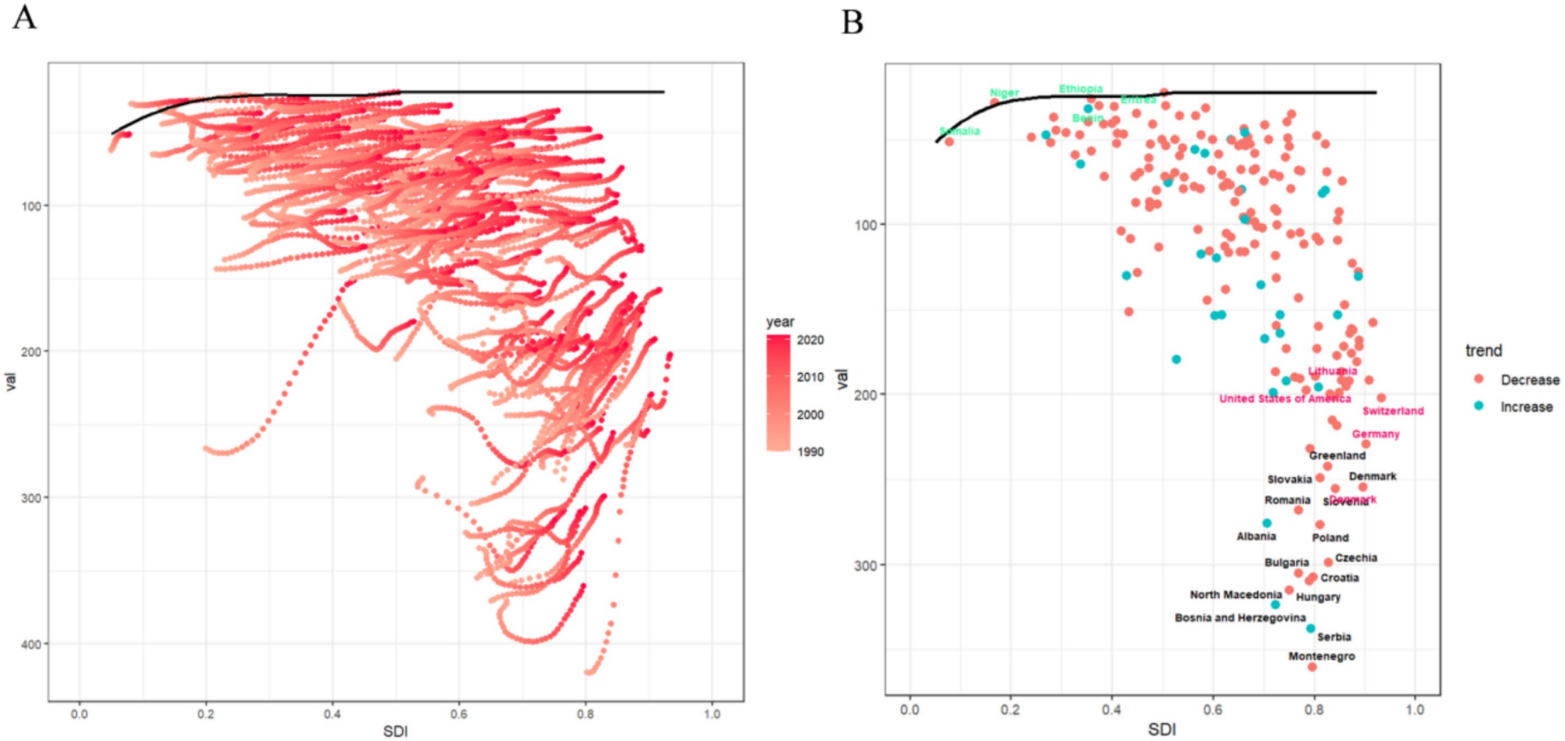
The frontier analysis results. **(A)** The frontier analysis based on ASDR and SDI from 1990 to 2021 is illustrated. The color gradient ranges from light pink (representing 1990) to dark pink (representing 2021). The boundaries are outlined with solid black lines. **(B)** The frontier analysis for 2021 based on ASDR and SDI is depicted. The boundaries are represented by solid black lines, and countries and regions are marked with dots. The top 15 countries and regions with the largest effective differences are highlighted in black. Border countries with low SDI and low effective variance are marked in green, while those with high SDI and relatively high effective variance are marked in red. Red dots indicate a decrease, and blue dots indicate an increase. ASDR, age-standardized DALYs rate; SDI, sociodemographic index.

This study utilizes global data from 1990 to 2021, focusing on ASDR and the SDI to assess the potential for health improvements across countries and regions at various development levels. Figure 6A illustrates the unrealized health gains over this period. The results indicate that, as socio-demographic indicators improve, the effective difference grows significantly. This suggests that countries with higher SDI have a higher potential to mitigate health burdens. We identified 15 countries with the highest potential for improvement: Montenegro, Serbia, Bosnia and Herzegovina, North Macedonia, Hungary, Croatia, Bulgaria, Czechia, Poland, Albania, Romania, Slovenia, Denmark, Slovakia, and Greenland. These countries have higher ASDR values compared to similar countries, highlighting considerable room for health improvements. In low-SDI countries (SDI < 0.5) such as Niger, Somalia, Ethiopia, Benin, and Eritrea, significant progress has been made in mitigating disease burdens. However, some high SDI countries (SDI > 0.85), including Denmark, Germany, Switzerland, the United States, and Lithuania, continue to show high effective differences, particularly in managing smoking-related LBP. This necessitates further improvements in these countries (see Figure 6B; Supplementary Tables 2–6).

### 3.5 Global prediction of the burden of LBP caused by smoking

**Figure 7:**
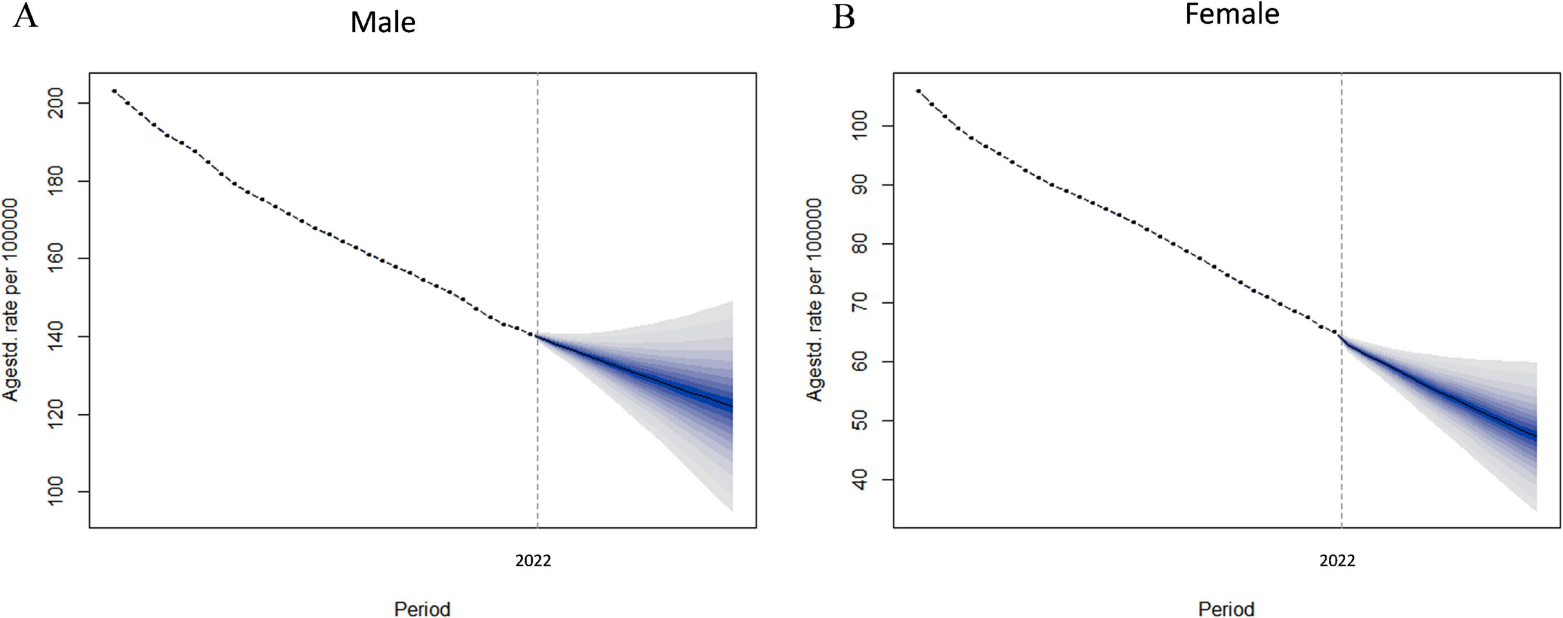
Analyzing the observed and predicted trends of ASDR of LBP attributable to smoking globally using the BAPC model. **(A)** ASDR of male. **(B)** ASDR of female. ASDR, age-standardized DALYs rate; LBP, low back pain; BAPC, Bayesian age-period-cohort.

This study is based on data from the GBD and utilizes the BAPC model for predictive analysis. The findings suggest that, from 2022 to 2036, the ASDR due to smoking-related LBP is expected to decline significantly in both males and females. By 2036, the global ASDR for smoking-related LBP in males is projected to reach 122.13 per 100,000 individuals (see Figure 7A), reflecting a 13.21% decrease from 2021. For females, the ASDR is expected to drop to 47.38 per 100,000 individuals by 2036 (see Figure 7B; Supplementary Table 7), representing a 27.25% reduction from 2021. This trend indicates that the burden of smoking-related LBP will be significantly reduced over the next decade, with females experiencing a greater decline than males, highlighting gender differences in disease burden improvement.

## 4 Discussion

Numerous studies have established that smoking is a significant risk factor for LBP (39, 40). Research indicates that smokers not only experience more intense pain but also suffer from it for longer periods compared to non-smokers (41). Additionally, the damage caused by smoking to LBP may be partially irreversible (42, 43). Smoking contributes to back pain through multiple mechanisms, including disc degeneration, inflammatory reactions, osteoporosis, diminished muscle function, psychological factors, and changes in gene expression. For example, smoking can increase the activity of matrix metalloproteinases (MMPs), which break down collagen and proteoglycans in the extracellular matrix of intervertebral discs, leading to disc structure damage (44). Moreover, smoking induces systemic inflammation, promoting the release of pro-inflammatory cytokines (e.g., IL-1β, TNF-*α*, and IL-6), which inflame the discs and surrounding tissues, exacerbating pain (45). Smoking also disrupts the balance between osteoblasts and osteoclasts, resulting in abnormal bone remodeling and further destabilizing the spine (46). Long-term smoking can impair neuromuscular control, increasing the likelihood of lumbar muscle strain (47). Recent research indicates that smoking may change the expression of genes linked to disc degeneration and pain through epigenetic mechanisms (e.g., DNA methylation), thereby impacting the development and progression of back pain (48). Furthermore, smokers may have heightened pain sensitivity, and smoking is frequently associated with psychological issues, including anxiety and depression, which may intensify pain sensitivity (49, 50). These findings highlight the strong connection between smoking and the underlying mechanisms of LBP.

The 2019 GBD study identified three main risk factors contributing to DALYs from LBP: smoking (15.7%), high body mass index (BMI, 6.7%), and occupational ergonomic factors (24%), with smoking ranking as the second-largest contributor. In regions with a high and middle-high SDI, smoking is the leading cause of LBP burden, particularly in Central Europe, where it accounts for 27.1% of cases. From a gender perspective, the LBP burden due to smoking is significantly greater in males than females, making smoking the primary risk factor for LBP among males in high and middle-high SDI regions. In 2019, the global number of current smokers aged 15 and older reached 1.14 billion (95% uncertainty interval: 1.13 billion–1.16 billion). The age-standardized smoking rate was 32.7% (32.3–33.0%) for males and 6.62% (6.43–6.83%) for females. That same year, global tobacco consumption totaled 7.41 trillion (95% uncertainty interval: 7.11 trillion–7.74 trillion) cigarette equivalents, translating to daily consumption of 20.30 billion (19.5 billion–21.2 billion) cigarette equivalents. China is the world’s largest consumer of tobacco, accounting for over one-third of global consumption, with 2.72 trillion (2.47 trillion–3.01 trillion) cigarette equivalents (51). This study provides the most up-to-date data on the burden of smoking-related LBP across 204 countries and regions from 1990 to 2021. The findings show that compared to 1990, the global DALYs of smoking-related LBP increased by 23.11% in 2021, while the ASDR dropped from 153.22 per 100,000 (95% uncertainty interval: 91.37–226.59) to 102.04 (95% uncertainty interval: 60.02–152.10), with an EAPC of −1.26%. This trend is partly due to population growth and epidemiological transitions. Notably, the DALYs of smoking-related LBP remain significantly greater in males than females, and the LBP burden due to smoking varies across regions and time, influenced by complex interactions with socio-demographic factors. The burden of smoking-related LBP is expected to continue declining in the future. However, strengthening tobacco control measures, particularly in regions with high smoking prevalence, remains essential to reduce the burden of this public health issue.

Previous research has shown that, from 1990 to 2021, smoking-related deaths increased by 25.43%, while DALYs rose by 15.64% for both males and females. Despite this, the age-standardized mortality rate (ASMR) and ASDR have shown a downward trend. The impact of smoking varies significantly between males and females. Males show notably higher smoking-related mortality and DALYs than females. Globally, the age-standardized smoking rate was 32.7% (95% uncertainty interval: 32.3–33.0%) for males and 6.62% (95% uncertainty interval: 6.43–6.83%) for females (52). This finding is consistent with our results: over the past 21 years, the ASDR for LBP due to high smoking declined for both males and females at similar rates. However, as of 2021, the ASDR in males (140.55 per 100,000) is still significantly higher than in females (65.13 per 100,000). A key factor contributing to these disparities is the significantly higher smoking prevalence among males, which is approximately eight times higher than that of females. This substantial gap results in a higher prevalence and severity of smoking-related health issues in men, with LBP being a common manifestation (53). Smoking increases the levels of carbon monoxide in the blood, which reduces the oxygen-carrying capacity of hemoglobin. This leads to hypoxia in intervertebral disc cells, impeding their normal metabolism and accelerating degeneration. Additionally, nicotine has been shown to exert direct cytotoxic effects on intervertebral disc cells, further exacerbating degeneration. Men, who have higher smoking rates and greater smoking intensity, consequently face a higher risk and greater severity of intervertebral disc degeneration (54). Smoking increases the levels of pro-inflammatory cytokines, which in turn activate the central nervous system and amplify pain signals, leading to exacerbated LBP. Despite the more robust immune activity in women, men’s higher smoking volume results in more severe inflammatory responses and more significant low back pain (55). Furthermore, specific genetic polymorphisms can render men more vulnerable to the detrimental effects of smoking. A case in point is the rs8040868 CT genotype of the CHRNA3 gene, which has been shown to be significantly correlated with the risk of lumbar disc herniation in the male population (56). One study showed that many more male students tried smoking (40.7%) than female students (20.6%). Also, more male students smoked over five cigarettes a day (7.2%) compared to just 1.0% of female students. These findings indicate that smoking behavior is more frequent and intensive among male students, thereby increasing their exposure to harmful substances (57). Psychologically, smoking is closely related to anxiety, depression, and other psychological factors. Research indicates that male smokers often exhibit stronger nicotine dependence than female smokers and show significant differences in motivation and behavior when it comes to quitting smoking. These findings suggest that men may be more prone to developing a stronger psychological dependence on smoking, which in turn can heighten their sensitivity to pain (58). Moreover, sociocultural factors are also of great significance. Research indicates that men are more reluctant to seek medical attention, which is closely associated with traditional masculine gender role concepts. This behavior results in men missing out on early disease warnings, thus increasing the risk of disease progression (59). These findings highlight the importance of considering gender differences when developing prevention and intervention strategies for smoking-related diseases.

Furthermore, we found that the burden of smoking-related LBP initially increases and then decreases with age. Between 1990 and 2021, crude DALY rates across all age groups saw a significant decline in all SDI regions. The highest number of DALYs was seen in the 40–44 age group, while the highest crude DALY rates was in the 60–64 age group. These results suggest that middle-aged individuals and younger older adult people experience a relatively higher burden of smoking-related LBP. The smoking prevalence and quantity of smoking are typically higher in the 40–44 age group. Individuals in this age group are often at the peak of their careers, facing substantial work pressure and frequent social activities, which makes smoking behavior more common and the quantity of smoking greater. The higher the quantity of smoking, the greater the risk of health problems caused by smoking, including LBP (60). Studies have demonstrated a clear dose–response relationship between the intensity of physical labor and the risk of developing chronic LBP. Notably, individuals within the 40–44 age group are more likely to be employed in jobs that involve either heavy physical labor or prolonged sitting, occupational factors that are known to elevate the risk of LBP (61). Individuals aged 40–44 often face significant life stressors, such as the dual burden of family and work responsibilities, which may lead to unhealthy lifestyles (e.g., lack of exercise, unbalanced diet). These factors, in conjunction with smoking, can increase the incidence of LBP. Although low back pain may begin at an earlier age, the 40–44 age group represents the early progression stage for many chronic diseases, such as intervertebral disc degeneration. Smoking accelerates the development of these diseases, leading to higher DALYs. Individuals in the 60–64 age group have often been exposed to smoking for several decades, during which the cumulative effects of smoking-induced chronic diseases, such as LBP, significantly increase. A study has indicated that in patients who have smoked for over a decade, the degeneration of the L3-L4 intervertebral disc is more pronounced, which is associated with the oxygen deprivation of the disc caused by smoking. At this age, many chronic diseases have reached advanced stages, leading to severe complications and higher disability rates. For example, smoking-induced LBP can further trigger disc herniation and spinal instability, which severely impact the quality of life (56). While the 40–44 age group has the highest number of DALYs, the 60–64 age group has a relatively smaller population size, resulting in a higher DALYs rate. In certain low- and middle-income countries, restricted access to healthcare among the older adult can result in untreated or inadequately treated diseases, thereby amplifying the overall disease burden (62).

The burden of smoking-related LBP varies significantly worldwide, which can be attributed to a range of factors. Data from 2021 reveal that the ASDR for smoking-related LBP differs by as much as 16 times between countries. In particular, some countries in Southeast Europe, including Montenegro, experience especially severe ASDR. This may be due to the high smoking prevalence, socio-economic conditions, cultural and social norms, and limited healthcare resources in these regions. Montenegro, like other parts of Southeast Europe, has a high smoking rate, lower levels of economic development, and limited healthcare resources, resulting in inadequate prevention and treatment of smoking-related diseases (63). Smoking is considered a social or cultural habit, particularly among males, and public awareness of its harmful effects remains insufficient (64). The GBD study indicates that between 1990 and 2021, more than 80% of countries saw a decline in disease burden, with Madagascar and Mexico experiencing the most significant declines. These improvements were likely driven by strict tobacco control measures, which include higher tobacco taxes, public smoking bans, advertising restrictions, and smoking cessation programs (65, 66). Health education campaigns also played a key role in increasing public awareness of smoking’s harms, specifically in preventing chronic conditions like LBP (67). Additionally, economic growth and enhanced healthcare resources strengthened the ability to prevent and treat smoking-related diseases (68). Regionally, Central Europe and high-income North America have higher-than-expected LBP burdens. At the national level, countries such as Montenegro, Serbia, Bosnia and Herzegovina, and Hungary also report higher-than-expected smoking-induced LBP burdens. These findings highlight the need for regions and countries to focus on implementing specific preventive and intervention strategies tailored to local characteristics. For example, Montenegro is a popular tourist destination. We can distribute informational materials at tourist attractions, hotels, and airports to raise awareness of the link between smoking and LBP. Community-based smoking cessation consultation points can be established to provide professional guidance and support. Additionally, when diagnosing back pain, hospital doctors should inquire about patients’ smoking history and recommend smoking cessation services. For patients who are motivated to quit smoking, nicotine replacement therapy and other supportive measures can be provided. Serbia has a well-developed industrial sector. Establishing smoke-free zones in factories and other industrial workplaces, conducting anti-smoking campaigns, and encouraging companies to offer smoking cessation incentives such as discounts on health check-ups and fitness subsidies are effective measures. Meanwhile, leveraging television, radio, and newspapers to air public service ads and conduct expert interviews will help raise public attention to this matter. Bosnia and Herzegovina can combine traditional medicine with modern smoking cessation methods, such as using herbal remedies and acupuncture to assist in quitting smoking and alleviating anxiety and discomfort. Moreover, working with religious communities to spread awareness about smoking cessation via religious sites and events is effective. Religious leaders can highlight the advantages of quitting smoking during their sermons and guide their congregations to engage in smoking cessation efforts.

This study conducted a national level frontier analysis using ASDR and SDI data. Among countries with a low SDI (<0.5), Niger, Somalia, Ethiopia, Benin, and Eritrea have demonstrated notable management capabilities. For example, Niger joined the WHO Framework Convention on Tobacco Control (WHO FCTC) in 2012, committing to comprehensive tobacco control measures (69). Despite ongoing political instability, Somalia’s transitional government passed the Tobacco Control Act in 2015, banning smoking in public places and restricting tobacco advertising (70). Ethiopia has actively launched nationwide campaigns to raise public awareness of smoking-related health risks, while Benin has effectively reduced tobacco consumption through higher tobacco taxes (71, 72). These countries have focused their efforts on legislative measures, tax policies, public education, and international cooperation. Their successes demonstrate that significant health improvements can be achieved through well-designed policies, even in resource-limited settings, offering valuable lessons for other low-income nations.

In contrast, countries with a high SDI (>0.85), such as Denmark, Germany, Switzerland, the United States, and Lithuania, show relatively higher effective differences concerning their development levels. In Denmark, smoking remains relatively common, especially among young people and low-income groups. According to 2020 data, the smoking prevalence among individuals aged 15 and older was approximately 16.2%, with rates of 18.4% among men and 18.7% among women (42). Despite the introduction of several tobacco control policies, including a nationwide smoking ban in 2007, Denmark’s policy contains multiple exemptions that limit its actual scope of coverage (73). Additionally, the rising popularity of e-cigarettes may have slowed the cessation of traditional smoking and attracted new nicotine users (74). In Germany, the tobacco industry invests approximately USD 112 million annually in advertising and marketing, primarily in cinemas and outdoor spaces, significantly undermining the impact of tobacco control policies (75). Switzerland has relatively lenient restrictions on tobacco advertising, particularly during international sports events, where tobacco-related promotions remain highly visible (76). In the United States, low-income populations and rural residents often struggle to access high-quality healthcare, contributing to a higher burden of smoking-related LBP (77). Meanwhile, the transition of Lithuania from a planned economy to a market economy has led to a significant increase in tobacco consumption, resulting in the persistence of smoking-related health issues (78). Addressing these issues requires the development of targeted tobacco control policies and health education initiatives tailored to each country’s specific circumstances. Denmark can further intensify its tobacco control measures targeting low socioeconomic groups to effectively reduce smoking rates. Imposing high taxes on heated tobacco products and e-cigarettes, and further increasing tobacco taxes, will not only effectively reduce smoking rates but also lower the risk of LBP caused by smoking. Moreover, Denmark should offer comprehensive smoking cessation support, such as nicotine replacement therapy and professional counseling, to assist smokers in quitting successfully. Germany can raise public awareness and enhance anti-smoking campaigns by publicizing the link between smoking and LBP through media and public health activities. In medical institutions, doctors should proactively inquire about patients’ smoking history and offer smoking cessation advice and treatment plans to smokers. Switzerland can conduct health education activities in schools and communities to raise public awareness of the dangers of smoking. Establishing smoking cessation consultation points in communities to offer professional guidance and support can assist smokers in quitting. Furthermore, Switzerland can develop personalized intervention plans by combining smoking cessation treatments with behavioral pain management strategies for smokers. The United States can cover smoking cessation services through medical insurance, enabling more smokers, especially those with low incomes and residents in rural areas, to access support for quitting smoking. Lithuania can draw on the successful experiences of other countries, such as Niger, to further strengthen its tobacco control legislation, for example by increasing tobacco taxes and expanding smoke-free areas. Through these targeted interventions, countries can effectively reduce the problem of LBP caused by smoking, thereby significantly improving public health levels.

This study has several limitations that should be considered when interpreting the results. First, there are inconsistencies in data sources and quality. The GBD study relies on data from epidemiological surveys, hospital records, and mortality registries across different countries, but the quality and scope of these data vary significantly. Particularly, data from low- and middle-income countries may be incomplete or biased. In low-income countries, due to insufficient data collection, smoking rates are underestimated. This can lead to an underestimation of the global burden of back pain caused by smoking, thereby affecting the allocation of global public health resources. To improve this situation, it is crucial to enhance data collection infrastructure, extend the reach of data collection, improve data quality, strengthen international cooperation, increase public involvement, secure policy and financial support, and carry out sensitivity analysis. These actions will boost the accuracy and reliability of research, providing a more robust basis for the formulation of global public health policies. Second, the causal relationship between smoking and LBP is complex. While smoking is recognized as a risk factor for LBP, the specific mechanisms (e.g., intervertebral disc degeneration and inflammatory responses) are not yet fully understood. Third, there are limitations in measuring smoking exposure. The GBD study typically uses smoking prevalence or quantity as exposure indicators, which may not fully capture the cumulative effects of smoking (such as smoking duration and depth of inhalation). Finally, the study employs a BAPC model to forecast the future burden of low back pain related to smoking but does not adequately address the limitations of the model. Future research could introduce interaction terms into the BAPC model, allowing for interactions between age, period, and cohort effects. Additionally, considering the use of nonlinear Bayesian models instead of the conventional linear BAPC models could be beneficial. Performing cross-validation, which involves dividing the data into training and testing sets, can assess the model’s predictive power across different subsets and determine if the model is overfitting or underfitting.

## 5 Conclusion

Overall, smoking remains a significant risk factor for LBP. From 1990 to 2021, the ASDR of smoking-induced LBP has shown a global downward trend, which is projected to continue in the future. However, despite the decline in age-standardized prevalence, data from the GBD study indicate that the absolute burden still increases due to population aging. Therefore, strengthening tobacco control policies, enhancing public health awareness, and implementing targeted prevention and intervention strategies in areas with high smoking rates and low socio-economic status will be essential moving forward to further reduce the burden of smoking-related LBP.

## Data Availability

The original contributions presented in the study are included in the article/Supplementary material, further inquiries can be directed to the corresponding author.

## Footnotes

^1^ ghdx.healthdata.org/gbd-results-tool
