# Supplementary figures and images for: Global burden of low back pain attributable to smoking in 204 countries and territories in 1990–2021
Source: Front Public Health. 2025 Jul 10;13:1584659. doi: 10.3389/fpubh.2025.1584659 (PMC12287117; doi:10.3389/fpubh.2025.1584659)

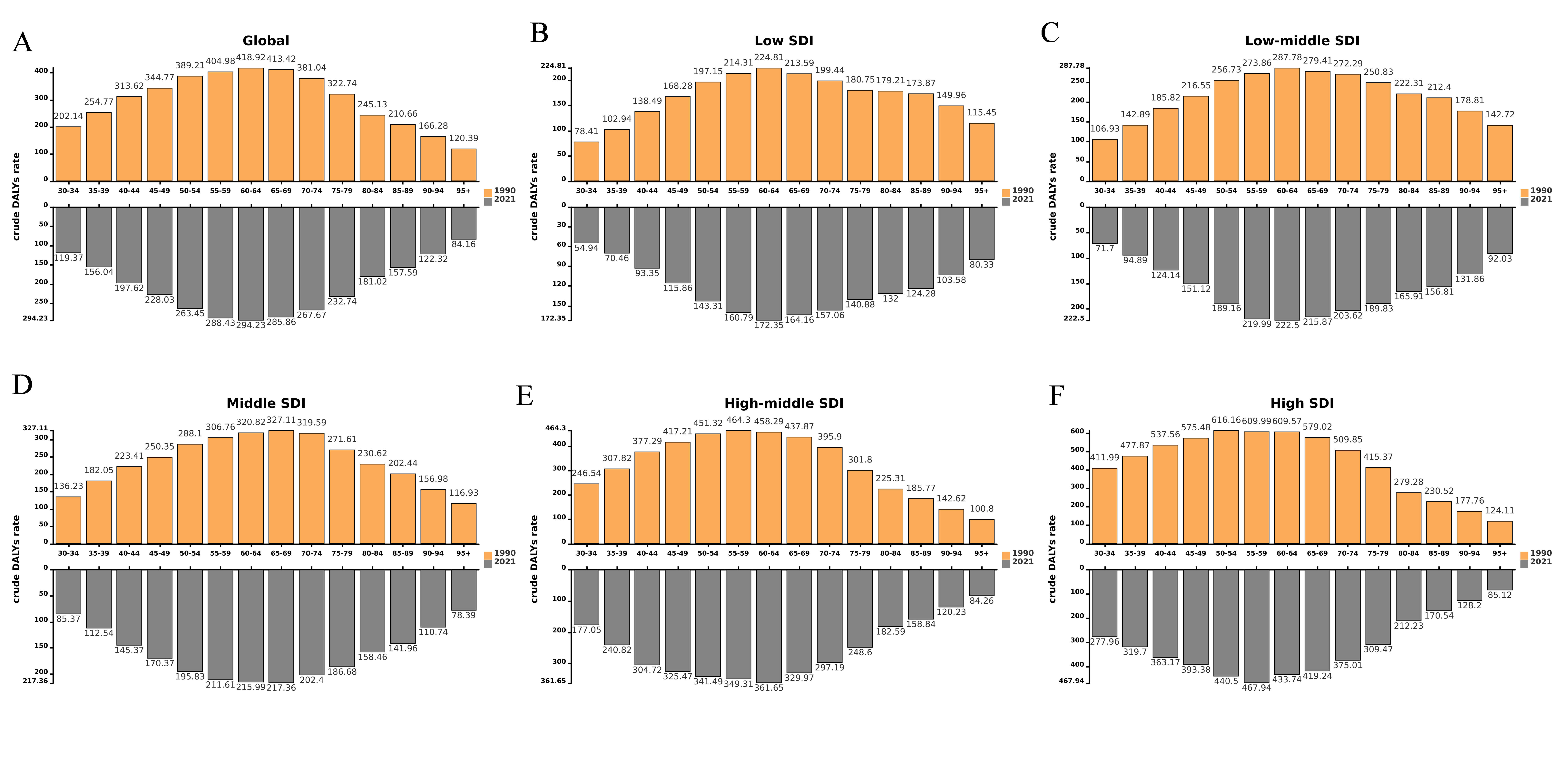

Supplement: Supplementary file 8 [file Image_1.tif]
